# Comparative Analyses of Two Established Scores to Assess the Stability of Spinal Bone Metastases Before and After Palliative Radiotherapy

**DOI:** 10.3389/fonc.2021.753768

**Published:** 2021-10-19

**Authors:** Tilman Bostel, Sati Akbaba, Daniel Wollschläger, Tristan Klodt, Laura Oebel, Arnulf Mayer, Sophia Drabke, Tanja Sprave, Jürgen Debus, Robert Förster, Harald Rief, Alexander Rühle, Anca-Ligia Grosu, Heinz Schmidberger, Nils H. Nicolay

**Affiliations:** ^1^ Department of Radiation Oncology and Radiation Therapy, University Medical Center Mainz, Mainz, Germany; ^2^ German Cancer Consortium (DKTK) Partner Site Mainz, German Cancer Research Center, Heidelberg, Germany; ^3^ Institute of Medical Biostatistics, Epidemiology and Informatics (IMBEI), University Medical Center Mainz, Mainz, Germany; ^4^ Department of Radiation Oncology, University Hospital of Freiburg, Freiburg, Germany; ^5^ German Cancer Consortium (DKTK) Partner Site Freiburg, German Cancer Research Center, Heidelberg, Germany; ^6^ Department of Radiation Oncology, University Hospital of Heidelberg, Heidelberg, Germany; ^7^ German Cancer Consortium (DKTK) Partner Site Heidelberg, German Cancer Research Center, Heidelberg, Germany; ^8^ Institute of Radiation Oncology, Cantonal Hospital Winterthur, University of Zurich, Winterthur, Switzerland; ^9^ Radiation Therapy Practice Bonn-Rhein-Sieg, Bad Godesberg site, Bonn, Germany

**Keywords:** spinal bone metastases, instability, radiotherapy, SINS, skeletal-related events

## Abstract

**Background and Purpose:**

To compare two validated spinal instability scores regarding the stabilizing effects and skeletal-related events (SREs) of palliative radiotherapy (RT) in patients with spinal bone metastases (SBM).

**Materials and Methods:**

Two hundred eighty-two osteolytic SBM of lung or breast cancer patients were analyzed for stability before and following RT based on the Spinal Instability Neoplastic Score (SINS) or the Taneichi score. Score concordance was quantified by absolute agreement and Cohen’s kappa coefficient. SREs were defined as fractures or local progression after RT. OS was quantified as the time between the start of RT and death from any cause.

**Results:**

At 3 and 6 months after RT, 35 and 50% of initially unstable SBM were re-stabilized according to SINS in patients still alive. Corresponding Taneichi score-based stabilization proportions were 25 and 46%, respectively. Comparison of both stability scores showed high absolute agreement for all time-points (range 71–78%, kappa range 0.35–0.44). SRE occurred more frequently in initially unstable SBM compared to stable SBM according to SINS (14 *vs.* 5%), but no such association could be shown for the Taneichi-based instability criterion. Poor general condition of patients was negatively associated with SINS-measured re-stabilization after 6 months, but no predictive factor for re-stabilization could be found for the Taneichi score.

**Conclusions:**

Despite the relatively high agreement between both stabilization scores, the SINS should be considered the standard for future studies on the stabilization effects of RT in SBM.

## Introduction

Spinal bone metastases (SBM) occur in up to 80% of patients with advanced solid tumors (1, 2). Affected patients often suffer from severe pain, movement restrictions, and/or neurological deficits. Radiotherapy (RT) is a key treatment for symptomatic SBM (3). In addition to the elimination of pain symptoms and restoration of skeletal function, instability of SBM with impending or manifest fractures represents another frequent indication for palliative RT. Unstable SBM not only affect spinal statics but may also threaten the integrity of the spinal cord and the branching nerves with potentially significant negative impact on the patients’ quality of life (4, 5). However, stability assessment of the spinal column is a major challenge in clinical practice and is often only carried out on the basis of clinical experience. This may result in under- or overdiagnosis of spinal instability, making communication between physicians of different disciplines very difficult, and leading to inconsistent therapeutic approaches. For this reason, the Spinal Instability Neoplastic Score (SINS) was introduced in 2010 by the Spinal Oncology Study Group and has since become the most adopted stability score for assessing SBM (6). Based on six categories, the SINS is a highly reliable tool to classify the stability of metastatically affected vertebral bodies into stable, potentially unstable, and unstable lesions (7). For unstable vertebral bodies, the SINS gives a clear recommendation for a stabilizing surgery and postoperative RT. In the case of potentially unstable vertebral body metastases, palliative RT is often preferred to surgery, especially for patients with poor prognosis. Unfortunately, there are only very limited SINS-based data on the effect of RT in unstable SBM (8). Instead, the data available so far are mainly based on the Taneichi score, which has also been validated (9–15).

Therefore, this retrospective study aimed to assess pre- and post-RT stability of spinal metastases using the SINS and Taneichi score, in order to verify their agreement, establish potential predictive factors for stability, and analyze skeletal-related events (SRE) and overall survival (OS) following RT.

## Material and Methods

### Patient Selection

A total of 221 patients with a median age of 63 years (range 34–88 years) and osteolytic SBM of the thoracic or lumbar spine with underlying breast (38%) or bronchial carcinoma (62%) were included in this retrospective study. All patients received one or more palliative RT at the University Hospitals of Mainz and Heidelberg between 2006 and 2012. The required patient data were taken from the medical records and cancer registers of the participating centers. The diagnosis of SBM was based on imaging techniques such as CT, MRI, or bone scintigraphy. As inclusion criteria, the spinal metastases had to have an osteolytic phenotype and be located in the thoracic or lumbar spine. This analysis has been approved by the independent ethics committees of the medical faculties of the universities of Heidelberg and Mainz (Heidelberg: S-513/2012, Mainz: 2020-15282).

### Stability Assessment

At baseline, as well as 3 and 6 months after palliative RT, the stability of metastatically affected vertebral bodies was assessed by CT imaging using the SINS and Taneichi scores. The Taneichi score is only validated for osteolytic bone metastases in the thoracic and lumbar spinal column. It is based purely on radiological criteria (degree of vertebral body destruction, involvement of the costovertebral joint and/or pedicle) to identify spinal lesions that have a very high risk of impending vertebral body collapse. The SINS, on the other hand, is validated for bone metastases of any phenotype throughout the entire spinal column. It is based on six criteria (location, type of pain, type of lesions, spinal alignment, presence of vertebral compression fractures, affection of posterolateral elements) to classify the affected vertebral bodies as stable, potentially unstable, and unstable. In this analysis, the instability criteria of the original publication were used (6, 16), i.e., all vertebral body metastases that were classified as at least potentially unstable (≥7 points) according to SINS or had a fracture risk of at least 50% according to Taneichi Score were evaluated as unstable. For both scores, the shift in stability from (potentially) unstable to stable and from stable to (potentially) unstable was independently determined by a board-certified radiologist in patients still alive at the time of evaluation. Furthermore, SREs after palliative RT were assessed, defined as new fractures or progressive sintering of SBM-affected vertebral bodies, or the need for re-irradiation. In the case of multiple bone metastases in a vertebral body or within the target volume, only the most severe lesion was evaluated. If several spinal regions were irradiated in a given patient, each region was evaluated separately in our analysis. A pain response was documented based on reduction of ≥2 points on the 10-point visual analogue scale according to the international consensus criteria (17). For partial and complete pain response to RT, the SINS criterion *“type of pain”* was rated with 1 and 0 points in our analysis, respectively.

### Treatment

Radiation treatment planning was based on planning CT examinations and, in the case of paravertebral tumor spread, supplemented with MRI scans. The radiation dose was administered *via* one or more dorsal or oblique dorsal photon fields (6 or 18 MV photon energy). The planning target volume (PTV) included the metastatically affected vertebral body or bodies and the adjacent intervertebral discs, and in most cases also the caudally and cranially adjacent vertebral bodies. Palliative RT was indicated if SBM caused pain symptoms, spinal instability, or neurological deficits. None of the patients in this analysis received additional surgery or other invasive procedures.

### Statistical Analysis

Overall survival (OS) was defined as the period from the beginning of first RT until death from any cause and estimated using the Kaplan-Meier method. Group differences in OS after first RT were assessed using the log-rank test. Logistic regression using generalized estimating equations was used to test whether the probability of stable lesions according to SINS and Taneichi score changed from baseline to 3 and 6 months after RT. Logistic regression using generalized estimating equations was used to test the association between the occurrence of any SRE and baseline instability of SBM. Association of prognostic factors “age at RT”, “tumor histology”, “KPS <70 *vs.* ≥70”, and “fractures prior to RT” with the SINS and Taneichi scores at 3 and 6 months post-RT was tested using separate univariate mixed ordinal logistic regression models. The concordance of the SINS and Taneichi scores in the patient cohort was checked using absolute agreement, and with Cohen’s kappa coefficient. Statistical analysis was done using the R statistical environment, version 4.0.2 (R Core Team 2020, Vienna, Austria). P values of p < 0.05 were considered statistically significant.

## Results

A total of 221 patients with 282 target volumes and 792 SBM (range 1–14 metastases per patient) of lung and breast carcinomas that were treated with palliative RT were assessed according to SINS and Taneichi score. Median follow-up after RT was 10.9 months (range 0.1–100.6 months). Further detailed information on patient and treatment characteristics is provided in **Table 1**.

**Table 1:** Patients’ and treatment characteristics.

Characteristics	Value	Percent
**Age (y)**		
Median	63	
Range	34–88	
**Gender (n)**		
Female	124	56.1
Male	97	43.9
**KPS (nRTc)**		
100	5	2.0
90	41	16.6
80	78	31.6
70	74	30.0
60	36	14.6
50	9	3.6
40	3	1.2
30	1	0.4
**Number of bone metastases (nRTvol)**		
Median	2	
Range	1–14	
Solitary	130	46.1
Multiple	152	53.9
**Spine involvement (nRTvol)**		
Thoracic	139	49.3
Thoracolumbar	66	23.4
Lumbar	77	27.3
**Primary tumor (n)**		
Breast carcinoma	83	37.6
NSCLC	126	57.0
SCLC	12	5.4
**Distant extraskeletal metastases (n)**		
Brain	29	13.1
Lung	33	14.9
Liver	38	17.2
Adrenal glands	15	6.8
Visceral	84	38.0
Other locations	15	6.8
**Single radiation dose (Gy)**		
Median	3	
Range	2–4	
**Cumulative dose (Gy)**		
Median	30	
Range	8–40	
**Fractionation of RT (nRTvol)**		
20 × 2.0 Gy	31	11.0
10 × 2.5 Gy	1	0.4
14 × 2.5 Gy	27	9.6
15 × 2.5 Gy	1	0.4
1 × 3.0 Gy	1	0.4
3 × 3.0 Gy	2	0.7
5 × 3.0 Gy	1	0.4
7 × 3.0 Gy	1	0.4
9 × 3.0 Gy	1	0.4
10 × 3.0 Gy	208	73.8
11 × 3.0 Gy	1	0.4
12 × 3.0 Gy	3	1.1
2 × 4.0 Gy	1	0.4
5 × 4.0 Gy	3	1.1
**Indications for RT**		
Pain (nRTvol)	235	83.3
Instability (nRTvol according to SINS)	218	77.3
Neurologic deficit (nRTc)	8	3.2
**Chemotherapy (n)**	150	60.7
**Other treatments for bone metastases**		
Orthopedic corset (nRTvol)	151	53.5
Bisphosphonates (nRTc)	217	87.9

KPS, Karnofsky performance score; y, years; RT, radiotherapy; Gy, Gray; n, number of patients (in total 221); nRTvol, number of RT volumes (in total 282); nRTc, number of RT courses (in total 247).

### Stability Assessment

Most patients exhibited unstable SBM prior to RT according to the SINS (217/282 SBM; 77%) and Taneichi score (224/282 SBM; 79%), respectively. The majority of these lesions (SINS: 88%, Taneichi: 82%) were associated with pain before the start of irradiation. In patients still alive at 3 and 6 months after RT, the change from baseline in the proportion of stable SBM was statistically significant (p < 0.001) at each time point with 35% (50/143) and 50% (52/104) of the primarily unstable SBM becoming stabilized according to SINS. Our analysis showed no statistically significant differences in the stabilization proportion between SBM of lung and breast cancer patients (see **Tables 2**, **3A**, and **3B**).

**Table 2:** Radiogenic changes in vertebral body stability according to SINS and Taneichi Score.

Stability assessment of SBMs	SINS	Taneichi score
**Prior to RT** *(total study population)*		
- stable (%)	64 (23%)	58 (21%)
- unstable (%)	218 (77%)	224 (79%)
**Shift from unstable to stable 3 mo. after RT** *(only SBM of the surviving patients in the entire study population)*		
- stable (%)	50 (35%)	39 (25%)
- unchanged unstable (%)	93 (65%)	115 (75%)
**Shift from stable to unstable 3 mo. after RT** *(only SBM of the surviving patients in the entire study population)*		
- unchanged stable (%)	40 (95%)	29 (94%)
- unstable (%)	2 (5%)	2 (6%)
**Shift from unstable to stable 6 mo. after RT** *(only SBM of the surviving patients in the entire study population)*		
- stable (%)	52 (50%)	53 (46%)
- unchanged unstable (%)	52 (50%)	62 (54%)
**Shift from stable to unstable 6 mo. after RT** *(only SBM of the surviving patients in the entire study population)*		
- unchanged stable (%)	29 (97%)	20 (100%)
- unstable (%)	1 (3%)	0 (0%)
**Shift from unstable to stable 6 mo. after RT** *(only SBM of the surviving breast cancer patients)*		
- stable (%)	40 (51%)	39 (48%)
- unchanged unstable (%)	39 (49%)	42 (52%)
**Shift from unstable to stable 6 mo. after RT** *(only SBM of the surviving lung cancer patients)*		
- stable (%)	12 (48%)	14 (41%)
- unchanged unstable (%)	13 (52%)	20 (59%)

SBM, spinal bone metastases; SINS, Spine Instability Neoplastic Score; RT, radiotherapy; NA, not analyzable, because the follow-up examination was missing due to a deterioration of the general condition or death.

**Table 3A:** Univariate analysis of prognostic factors related to stabilization of initially unstable SBM according to SINS.

Predictor	3 months after RT			6 months after RT		
	p-value	OR	CI	p-value	OR	CI
Age	0.99	1.001	0.941–1.064	0.80	0.995	0.954–1.037
Lung cancer (*vs.* breast cancer)	0.41	1.829	0.434–7.713	0.16	2.196	0.742–6.500
KPS (<70% *vs.* ≥70%)	0.20	0.273	0.037–1.996	0.02	0.158	0.032–0.790
Fractures before RT (yes *vs.* no)	0.88	0.919	0.294–2.868	0.73	0.850	0.337–2.142

**Table 3B:** Univariate analysis of prognostic factors related to stabilization of initially unstable SBM according to Taneichi score.

Predictor	3 months after RT			6 months after RT		
	p-value	OR	CI	p-value	OR	CI
Age	NA	1.006	NA	0.65	1.008	0.975–1.042
Lung cancer (*vs.* breast cancer)	0.09	1.537	0.929–2.543	0.21	1.717	0.740–3.983
KPS (<70% *vs.* ≥70%)	0.63	0.845	0.424–1.683	0.69	0.775	0.223–2.698
Fractures before RT (yes *vs.* no)	0.60	0.874	0.527–1.448	0.58	0.805	0.373–1.736

Prior to RT, the average SINS was 8.3 [standard deviation (SD) 2.3, range 3–15]. After palliative RT, the average SINS decreased to 6.7 (SD 2.4, range 2–12) after 3 months and to 6.0 (SD 2.2, range 1–12) after 6 months. According to the Taneichi score, the corresponding 3- and 6-month stabilization proportions were 25% (39/154 SBM) and 46% (53/115 SBM), respectively, representing a statistically significant change from baseline (p < 0.001 for each time point; see **Table 4**). The exact distribution of Taneichi scores before and after palliative RT is summarized in detail in **Table 4**.

**Table 4:** Stability assessment of irradiated SBM before and after palliative RT according to the Taneichi score.

Stability assessment of irradiated SBM	n (%)
**Taneichi classification prior to RT**	
- A	31 (11.0)
- B	48 (17.0)
- C	32 (11.3)
- D	55 (19.5)
- E	67 (23.8)
- F	47 (16.7)
- G	2 (0.7)
**Taneichi classification 3 months after RT**	
- A	55 (29.7)
- B	29 (15.7)
- C	19 (10.3)
- D	25 (13.5)
- E	35 (18.9)
- F	21 (11.4)
- G	1 (0.5)
**Taneichi classification 6 months after RT**	
- A	62 (45.9)
- B	20 (14.8)
- C	9 (6.7)
- D	17 (12.6)
- E	15 (11.1)
- F	11 (8.1)
- G	1 (0.7)

SBM, spinal bone metastases; RT, radiotherapy; n, number of patients.

Our analysis showed only slightly different 3- and 6-month stabilization proportions of SBM in breast and lung cancer patients who were still alive at the time of evaluation (see **Table 2**). Taking into account patients who died, the SINS-based stabilization proportions of SBM in the entire study population at 3 and 6 months after palliative RT were only 23% (50/217) and 24% (52/217), respectively. The corresponding Taneichi-based stabilization proportions were 17% (39/224) and 24% (53/224). The different survival prognosis of breast and lung cancer patients had a substantial impact on stabilization probability of primary unstable SBM. In breast cancer patients, the 6-month stabilization probability was 43% (40/93 SBM) and 41% (39/96 SBM) according to the SINS and Taneichi scores, while in lung cancer patients the corresponding values for both scores were only 10 and 11% (SINS: 12/124 SBM, Taneichi: 14/128 SBM).

In our analysis, the SINS criteria *“type of lesions”*, *“type of pain”,* and *“presence of vertebral compression fractures”* were decisive for the change in stability assessment of primary unstable SBM, while the scores in the other SINS criteria remained stable over time. Regarding the *“type of lesions”* criterion, recalcification was responsible for an improvement in the SINS stability category (i.e., shift from potentially unstable to stable) in 56% of initially unstable osteolytic SBM 3 months after RT (28/50; mixed type of lesions 50%, blastic lesions 6%, no recalcification 44%) and 81% of SBM 6 months after RT (42/52; mixed type of lesions 62%, blastic lesions 19%, no recalcification 19%), respectively. Regarding the *“type of pain”* criterion, an improvement of the stability due to an RT-induced pain response was present in 75% of symptomatic primary unstable SBM in our analysis (117/157; partial response 40%, complete response 35%).

A shift in stability from stable to (potentially) unstable according to both scores was only rarely observed following RT (see **Table 2**). In SINS, a Karnofsky Performance Score of less than 70% had a statistically significant association with worse stabilization probability in the univariate mixed ordinal logistic regression for patients still alive 6 months after palliative RT (see **Table 3A**); in contrast, no predictive factors could be identified for the Taneichi Score in patients still alive 3 and 6 months after palliative RT that could prospectively predict stabilization of primarily unstable SBM (see **Table 3B**).

When comparing the two stability scores, absolute agreement both before and at 3 and 6 months after RT was high (78, 71, and 73%, respectively), but Cohen’s kappa coefficients were low due to inhomogeneous marginal frequencies (0.35, 0,41 and 0,44, respectively).

### Skeletal-Related Events

Pathologic fractures were detected in 38% of SBM prior to RT (106/282). Up to 6 months after RT, new fractures and progressive sintering of pre-existing fractures within the vertebral bodies were observed in 3% (8/282) and 8% of SBM (22/282), respectively. According to SINS and Taneichi Score, the majority of these post-RT fractures were already initially assessed unstable (90 and 87%, respectively).

Most patients with post-RT fractures received osteoprotective therapy with bisphosphonates or RANK ligand inhibitors (90%) and had already been provided with a corset (66%) before the fracture. Associated pain was reported in 67% of post-RT fractures (20/30 SBM). In addition to post-RT fractures, three SBMs were locally progressive at follow-up with a need for re-irradiation due to new neurological deficits, resulting in an overall SRE proportion of 12% (33/282 target volumes). The presence of an initial pathological fracture in the irradiated area resulted in increased SRE proportions compared to unfractured metastatic lesions (21 *vs.* 6%). Furthermore, SINS-based initial vertebral body instability was significantly associated with the occurrence of SRE after palliative RT (p = 0.046, OR 3.38, 95% CI 1.02–11.22, Wald test), which was the case in 14% of primary unstable SBM (30/217) compared to only 5% of primary stable SBM (3/65). In contrast, this association could not be shown for the Taneichi-based instability criterion (p = 0.22, OR 1.97, 95% CI 0.66–5.81, Wald test).

### Overall Survival

**Figure 1:**
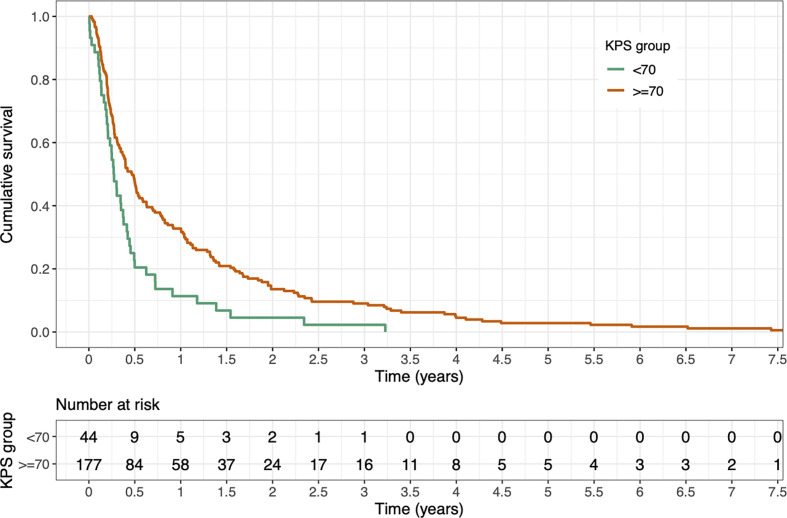
Kaplan-Meier estimate of overall survival (OS) after first radiotherapy stratified by Karnofsky Performance Score (KPS) <70 *vs.* ≥70. OS was significantly better for patients with a KPS of ≥70% (p < 0.001, log-rank test).

For the entire study population, median OS after first palliative RT of SBM amounted to 4.8 months (95% CI 3.8–6.0 months); the corresponding 6-, 12-, and 24-month OS was 42% (95% CI 36–49%), 29% (95% CI 23–35%), and 12% (95% CI 8–17%), respectively. Our analysis showed significantly worse OS after first RT for patients with Karnofsky Performance Scores below 70% compared to patients with scores of ≥70% (p < 0.001) (see **Figure 1**).

**Figure 2:**
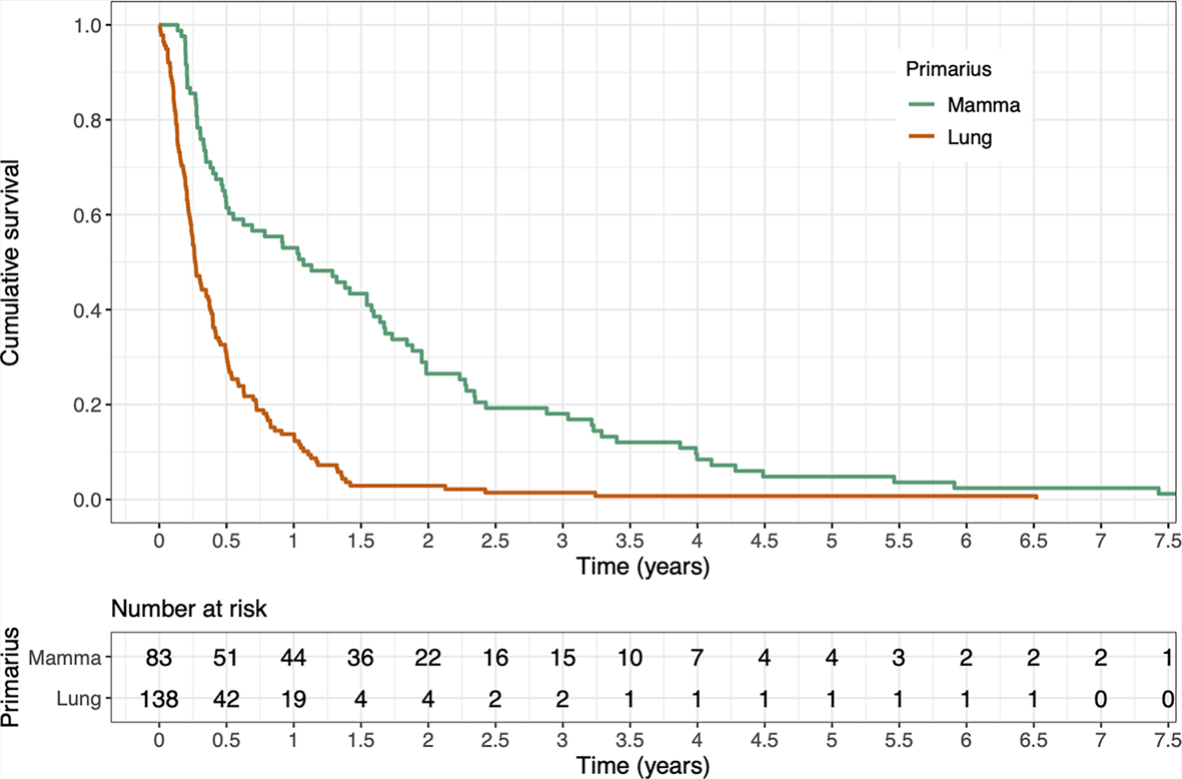
Kaplan-Meier estimate of overall survival after first radiotherapy stratified by tumor histology. OS was significantly better for breast cancer patients compared to lung cancer patients (p < 0.001, log-rank test).

Tumor histology was significantly associated with OS after palliative RT of SBM, with breast cancer patients having a considerably better prognosis than lung cancer patients (p < 0.001, median OS 12.9 months *vs.* 3.2 months) (see **Figure 2**).

## Discussion

Vertebral instability of SBM represents a key indication for palliative RT, which aims to support recalcification and to improve bone stability. Despite the clinical significance, data on RT-induced stabilization of primarily unstable SBM are limited (18). In particular, the role of stability scores in the context of assessing radiation-induced stabilization remains largely unknown (8).

In our dataset, palliative RT of primarily unstable SBM rarely lead to stabilization in lung cancer patients, whereas nearly half of unstable SBM of breast cancer patients re-stabilized within 6 months after RT. Regarding OS after RT, our evaluation showed significant differences between patients with osseous metastatic lung and breast cancer (median OS 12.9 months *vs.* 3.2 months). Consequently, the likelihood of stabilization of primarily unstable SBM depends to a large extent on the prognosis of the patients, as recalcification of osteolytic SBM is not expected before 3–6 months after palliative RT (19).

Thus, the relevance of stabilization of unstable SBM is higher for patients with a median OS exceeding 6 months. In our study, tumor histology and the Karnofsky Performance Score were found to be the crucial prognostic factors for OS, which is consistent with the results of previous studies predicting OS in patients with bone metastases (20, 21). Differences in recalcification and stabilization rates between different tumor entities are to a large extent due to the different prognoses, but other factors may also play important roles, such as the individual radiation sensitivity of the respective tumor cell types, the individual tumor microenvironment, the radiation dose or simultaneous systemic treatments. To date, the exact underlying mechanism of radiation-induced recalcification of osteolytic bone metastases remains incompletely understood.

For patients with a low chance of achieving bone stability 6 months after conventional palliative RT, alternative approaches to improve metastatic spinal stability may be considered. For such situations, further dose escalation in the bone metastases through simultaneous integrated boost (i.e., hypofractionated ablative radiotherapy) or even stereotactic body radiotherapy (SBRT) may be promising options. Dose escalation strategies have the potential to improve stabilization in patients with unstable SBM, particularly in patients with good life expectancy and SBM from tumor entities with a relatively low chance of bone stability at 6 months or those with oligometastatic disease. However, potential benefits of SBRT must be balanced against the increased risk of side effects such as fractures or neurologic deficits (22, 23). Surgical stabilization options may also become more relevant to patients. However, compared to SBRT or conventional palliative RT, surgery may interrupt necessary systemic treatments for a considerable time due to perioperative comorbidities.

In assessing vertebral body stability, our analysis showed a relatively good agreement between the SINS and Taneichi scores, with slightly better stabilization rates as assessed by the SINS. This is primarily explained by the inclusion of clinical assessment parameters. Therefore, improved stability scores in our evaluation were also measured by the SINS in case of only a pain response in the absence of recalcification. Nevertheless, our study has shown that previously reported stabilizing effects of palliative RT, based on the Taneichi Score, can be transferred relatively well to the SINS (9, 11, 13, 14, 16, 18, 24). In spinally metastasized head-and-neck tumors, a recent publication assessed stability also on the basis of the SINS (8).

For the SINS, a Karnofsky Performance Score of less than 70% had a statistically significant negative association with the probability of stabilization in our analysis for patients still alive at 6 months after palliative RT. This can be potentially explained by a reduced physical activity of patients with reduced performance, which could negatively impact bony mineralization. In contrast to the results of a recently published study, we could not identify any predictive factors for the Taneichi score in patients still alive at 3 and 6 months after palliative RT that could predict stabilization of primarily unstable SBM (24).

In our study population, pathologic fractures of unstable SBM were shown to be a common clinical problem before starting irradiation. After palliative RT, SRE occurred in 12% of irradiated SBM due to new fractures (3%), progressive fractures (8%), and local progression with the need for re-irradiation (1%). Most post-RT fractures occurred in patients with initial fractures and unstable SBM, and two-thirds of patients with a post-RT fracture reported associated pain. In the literature, secondary fractures, spinal cord compression, and re-irradiation rates after palliative multifractional RT were reported in 2–5, 4–6, and 7–9% of cases, respectively (25–28). The higher number of fractures in our study population can be explained by the high proportion of initially complicated SBM that were not adequately considered in the landmark studies and large literature reviews (25–28).

The presence of a pathologic fracture prior to RT resulted in an increase of SRE in our analysis compared to unfractured metastatic bone (21 *vs.* 6%). Furthermore, SINS-based initial vertebral instability was found to be significantly associated with the occurrence of SRE, whereas no such association could be demonstrated for Taneichi-based vertebral instability.

Our study has some limitations, especially considering the retrospective character of this patient cohort. For instance, data on clinical factors that may influence bone stabilization and fracture probability such as, e.g., osteoporosis, medication or physical activity could not be systematically collected in our cohort. Furthermore, patients’ quality of life could not be assessed retrospectively and requires further prospective investigation. Our evaluation did not include cervical and sacral SBM, as the Taneichi Score is only validated for osteolytic thoracic and lumbar SBM.

For this analysis, we intentionally included only patients who had not received RT in recent years to exclude potential effects of modern systemic therapies such as immunotherapy on bony remineralization. Thus, as a result of improved survival, SRE and stabilization rates of patients with more recently treated unstable SBM may be higher than reported here. Therefore, the current analysis intends to serve as baseline examination for subsequent histology-specific stability analyses of SBM irradiated in recent years.

In summary, the choice of radiation dose and fractionation should take into account not only the therapeutic goal but also clinical factors such as patients’ general condition and comorbidities, the extent of metastatic disease, and the overall prognosis of the patients, since significant recalcification of osteolytic SBM can first be detected at 3–6 months after palliative RT. The results of our retrospective evaluation support the urgent need to initiate prospective studies to systematically assess irradiation effects and complication rates (SRE) as a function of initial SINS-based vertebral body instability in the era of modern systemic therapies.

## Conclusion

Our analysis showed a relatively high agreement between two widely available clinical stabilization scores. The data published so far based on the Taneichi score can therefore be transferred relatively well to the SINS. However, compared to the SINS, the Taneichi Score has some important limitations (i.e., validation only for osteolytic SBM and thoracolumbar lesions), so the SINS should be further considered in future studies on the stabilization effects and complications of palliative RT in SBM. In this regard, initial vertebral body instability according to SINS and pre-existing fractures seemed to increase the risk for SRE after RT.

## Data Availability Statement

The raw data supporting the conclusions of this article will be made available by the authors, without undue reservation.

## Ethics Statement

The studies involving human participants were reviewed and approved by the independent ethics committees of the medical faculties of the universities of Heidelberg and Mainz (Heidelberg: S-513/2012, Mainz: 2020-15282). Written informed consent for participation was not required for this study in accordance with the national legislation and the institutional requirements.

## Author Contributions

TB and SA equally contributed to this manuscript. TB, SA, and NN developed and planned the retrospective analysis. DW is responsible for statistical considerations/basis of the analysis. TB, SA, TK, LO, AM, SD, TS, JD, RF, HR, AR, A-LG, HS, and NN participated in data collection and/or interpretation of the results. TB wrote the manuscript. All authors contributed to the article and approved the submitted version.

## Conflict of Interest

The authors declare that the research was conducted in the absence of any commercial or financial relationships that could be construed as a potential conflict of interest.

## Publisher’s Note

All claims expressed in this article are solely those of the authors and do not necessarily represent those of their affiliated organizations, or those of the publisher, the editors and the reviewers. Any product that may be evaluated in this article, or claim that may be made by its manufacturer, is not guaranteed or endorsed by the publisher.
